# Synthesis and assessment of copper-based nanoparticles as a surface coating agent for antiviral properties against SARS-CoV-2

**DOI:** 10.1038/s41598-022-08766-0

**Published:** 2022-03-22

**Authors:** Agung Purniawan, Maria Inge Lusida, Royan Wafi Pujiyanto, Aldise Mareta Nastri, Adita Ayu Permanasari, Alfonsus Adrian Hadikusumo Harsono, Nur Hafidzah Oktavia, Sigit Tri Wicaksono, Jezzy Renova Dewantari, Rima Ratnanggana Prasetya, Krisnoadi Rahardjo, Mitsuhiro Nishimura, Yasuko Mori, Kazufumi Shimizu

**Affiliations:** 1 Department of Materials and Metallurgical Engineering, Institut Teknologi Sepuluh Nopember, Surabaya, Indonesia; 2 Research Center for Advanced Materials and Nanotechnology, Institut Teknologi Sepuluh Nopember, Surabaya, Indonesia; 3 Institute of Tropical Disease, Indonesia-Japan Collaborative Research Center for Emerging and Re-Emerging Infectious Diseases, Universitas Airlangga, Surabaya, Indonesia; 4 Center for Infectious Diseases, Kobe University Graduate School of Medicine, Kobe, Japan

**Keywords:** SARS-CoV-2, Viral infection, Nanoparticles

## Abstract

To halt the pandemic of severe acute respiratory syndrome coronavirus 2 (SARS-CoV-2), governments around the world have imposed policies, such as lockdowns, mandatory mask wearing, and social distancing. The application of disinfecting materials in shared public facilities can be an additional measure to control the spread of the virus. Copper is a prominent material with antibacterial and antiviral effects. In this study, we synthesized copper nanoparticles (CuNPs) as a surface coating agent and assessed their antiviral activity against SARS-CoV-2. CuNPs with a mean size of 254 nm in diameter were synthesized from copper sulfate as a source and were predominantly composed of copper oxide. The synthesized CuNPs were mixed with resin-based paint (CuNP/paint) and sprayed on the surface of stainless steel remnants. SARS-CoV-2 lost 97.8% infectivity on the CuNP/paint-coated surface after 30 min of exposure and more than 99.995% infectivity after 1 h of exposure. The inactivation rate was approximately 36-fold faster than that on the paint alone-coated and uncoated surfaces. The CuNP/paint-coated surface showed powerful inactivation of SARS-CoV-2 infectivity, although further study is needed to elucidate the inactivation mechanisms. Applications of CuNP/paint coatings to public or hospital facilities and other commonly touched areas are expected to be beneficial.

## Introduction

The outbreak of coronavirus disease 2019 (COVID-19) caused by severe acute respiratory syndrome coronavirus 2 (SARS-CoV-2), first recognized in Wuhan, China in late December 2019, has spread rapidly to the entire world. The World Health Organization officially declared the outbreak a global pandemic on 11 March 2020. Governments around the world have imposed policies, such as lockdowns, mandatory mask wearing, hand washing, and social distancing, to halt the pandemic. However, it has not yet been doused and has caused more than 120 million infections worldwide, with 2.2% fatality as of 16 March 2021^1^.

SARS-CoV-2 is an enveloped virus, the genome of which consists of a single-stranded RNA of nearly 30,000 nucleotides in a positive-sense^2^. Unlike influenza virus carrying RNA-dependent RNA polymerase (RdRP) inside the particle together with negative-sense RNA as the genome, SARS-CoV-2 virus particles do not carry RdRP inside and thus are assumed to survive quite a long time in the environment. It was reported that the virus could survive and stay infectious for up to 7 d among different surfaces, such as plastics, stainless steels, and cardboard^3–5^. Other studies reported environmental contamination of SARS-CoV-2 in healthcare premises^6,7^. Contaminated commonly used surfaces are mostly located in public or hospital facilities such as handrails, doorknobs, sinks, elevator buttons, light switches, and benches. The main route of virus transmission is through person to person^8^. There is also the possibility that the contaminant virus dried on surfaces is stirred up with dust^9^ and lands on respiratory mucus to start infection; it can serve as an indirect transmission route. Thus, the application of disinfecting materials on shared public facility surfaces can be an additional measure to control virus spreading.

Silver and copper are well known for their antibacterial and certain antiviral properties^10,11^. Utilization of nanomaterials has been applied for this purpose because as their size decreases, the available surface area dramatically increases, and consequently, there is an increase over their original properties^12^. Among them, silver nanoparticles (AgNPs) should be used with caution because of their cytotoxic effects and potential derangement of the environment^13,14^. Copper nanoparticles (CuNPs) can be a cheaper and safer alternative, with similar antiviral properties and wider availability^15^. However, replacing all the already installed materials in public facilities is not a reasonable option. Instead, coating the surface with CuNP/paint can provide an affordable solution. This study aims to establish a method to synthesize CuNPs and to assess CuNP/paint coating for antiviral activity against SARS-CoV-2.

## Materials and methods

This study aims to establish a method to synthesize CuNPs and to assess CuNP/paint coating for antiviral activity against SARS-CoV-2 using the following methods. The experiments, which involve the SARS-CoV-2 virus, have been performed in the BSL3 laboratory at the Institute of Tropical Disease, Airlangga University, Surabaya, Indonesia.

### Synthesis of copper nanoparticles (CuNPs)

CuNPs were synthesized using a two-step reduction process following the methods reported by Yang et al.^16,17^ and Wen et al.^18^. Briefly, 63 g copper sulfate (CuSO_4_) was dissolved in 300 mL of demineralized water, followed by heating to 45 °C and stirring at 200 rpm until completely dissolved. While stirring, ethylenediaminetetraacetate (EDTA, C_10_H_16_N_2_O_8_) was added to the solution. Then, the mixture was extracted with oleic acid using a separator funnel. Then, for the first reduction, glucose was added at 60 °C, and the mixture was stirred at 400 rpm. After the solution became homogenized, ascorbic acid was added to reduce Cu compounds at 60 °C with stirring at 400 rpm. Finally, the sediments appeared in the bottom of the beaker glass and were collected on a filter.

The synthesized CuNPs were mixed with acrylux AAC955 acrylic resin water-based paint (Propan, Tangerang, Indonesia) using a mechanical stirrer at 200 rpm for 5 min to ensure homogeneity spread. The material was sprayed onto stainless steel remnants to coat the surfaces. Surfaces coated by paint alone without CuNPs and uncoated surfaces were also prepared for comparison.

### Characterization of CuNPs

The products were analyzed by scanning electron microscopy energy-dispersive X-ray spectroscopy (SEM–EDX; FEI, USA) for morphology and energy-dispersive X-ray spectroscopy, by particle size analysis (PSA; Malvern, UK) for size distribution, and by X-ray diffraction (XRD; Malvern, UK) for phase composition and crystallinity.

### Cell culture

Vero cells (obtained from Laboratory of Dengue, Institute of Tropical Disease, Universitas Airlangga) were used in this study for titration of virus infectivity. Cells were cultured in Dulbecco’s modified Eagle’s medium (DMEM) containing 5% fetal bovine serum (FBS), 100 μg/mL penicillin and 100 μg/mL streptomycin at 37 °C in a 5% CO_2_ incubator. Cells at 100% confluence were used in the experiments.

### Virus preparation

An isolate of SARS-CoV-2 obtained in our laboratory (ITD-4859: GISAID EPI_ISL_529965; the Pango lineage: B.1.470) was propagated using Vero cells in 6-well plates (Corning, New York, USA). Each well was inoculated with 1 mL of the virus dilution at a multiplicity of infection of 0.01 and incubated at 37 °C in a 5% CO_2_ incubator. DMEM used for virus propagation contained 2.5 μg/mL trypsin (TPCK treated, Worthington Biochemical Corporation, USA) without FBS, which we recently found to enhance virus infectivity (Manuscript in preparation). After 2 d of incubation, culture fluids were harvested and clarified by centrifugation at 6,000 rpm for 5 min. The supernatant was mixed with a half volume of 0.6% bovine serum albumin (fraction V, modified Cohn, pH 5.2; Merck, Germany) in TGS (25 mM Tris–HCl, 140 mM NaCl, 5 mM KCl, 0.7 mM Na_2_HPO_4_-12H_2_O, 5.6 mM glucose) and stored at -80 °C until use.

### Measurement of virus infectivity in cell culture

Infectivity titration was carried out by a 50% tissue culture infectious dose (TCID_50_) assay using Vero cells in 96-well plates. Triplicates of 0.1 mL of serial tenfold dilutions of samples in DMEM containing trypsin at a concentration of 2.5 μg/mL were inoculated into 100% confluent monolayer cells. After incubation for 2 d, the culture fluids were tested by reverse transcriptase-polymerase chain reaction (RT-PCR) for virus propagation. The full spread of infection in a well was confirmed by microscopic observation of complete CPE (cytopathic effect; rounding of the cells and detaching from the bottom surface). TCID_50_ is determined using Reed and Muench method^19^.

### RT-PCR

Extraction of RNA was performed using an RNeasy kit (QIAGEN, Valencia, California). Detection of the viral genomes was performed with one-step TaqMan real-time RT-PCR using a one-step RT-PCR kit (Thunderbird, Toyobo, Japan). The reaction conditions were as follows: step 1, reverse transcription for 15 min at 50 °C; step 2, initial denaturation for 3 min at 95 °C to inactivate reverse transcriptase and to activate Taq polymerase; and step 3, 45 cycles of amplification for 5 s at 95 °C and 40 s at 60 °C. Primers and TaqMan probes were designed to detect the RdRP gene of SARS-CoV-2. The forward primer was GTGAR ATGGT CATGT GTGGC GG (nucleotides 15,431 to 15,452 of SARS-CoV-2 Wuhan-Hu-1, GenBank MN908947), the reverse primer was GTYTA CAATT TSTGT GATAA TCGAT (15,530 to 15,505), and the probe was CAGGT GGAAC CTCAT CAGGA GATGC (15,470 to 15,494). Viral RNA of 90-nucleotide (nt) length was synthesized as a positive control, the sequence of which was 15,431 to 15,530 of the virus genome lacking 15,459 to 15,468. The synthesized RNA was also used to generate a standard curve for one-step RT-PCR to convert cycle threshold (Ct) values obtained to RdRP RNA copy numbers. RT-PCR was performed using an Applied Biosystems 7500 Fast and its software (Applied Biosystems, Massachusetts, USA).

### Assays of the antiviral properties of CuNP/paint against SARS-CoV-2

The 2.5 × 2.5 cm^2^ stainless steel remnants with CuNP/paint coating, paint alone coating, and uncoating were prepared and placed in a well of 6-well plates one by one. An aliquot of 20 µL of virus dilution with 0.2% BSA/TGS was applied on the test surface and covered by a circular glass slip of 1.13 cm in diameter foaming a cylindrical shape of 1 cm^2^ base area by 0.2 mm height and placed inside a class II safety cabinet at 24 °C with relative humidity of 40–50% for various times. The virus suspensions were recovered in tubes by two washes with 90 µL of 0.2% BSA/TGS; thus, the treated virus was recovered in 200 µL. The recovered virus was titrated for infectivity in Vero cell culture and subjected to RT-PCR for RdRP RNA to estimate the copy number of the genomic RNA. The results were obtained from triplicate measurements and expressed as a calculated value per 20 µL of the exposed samples.

### Statistics

All data were expressed as means ± standard deviation. Statistical analysis was performed using the F test and Student’s t test functions of Microsoft Excel for Mac, version 14.5.9. A two-sided *P* < 0.05 was considered statistically significant.

## Results

### Characterization of synthesized CuNPs

**Figure 1:**
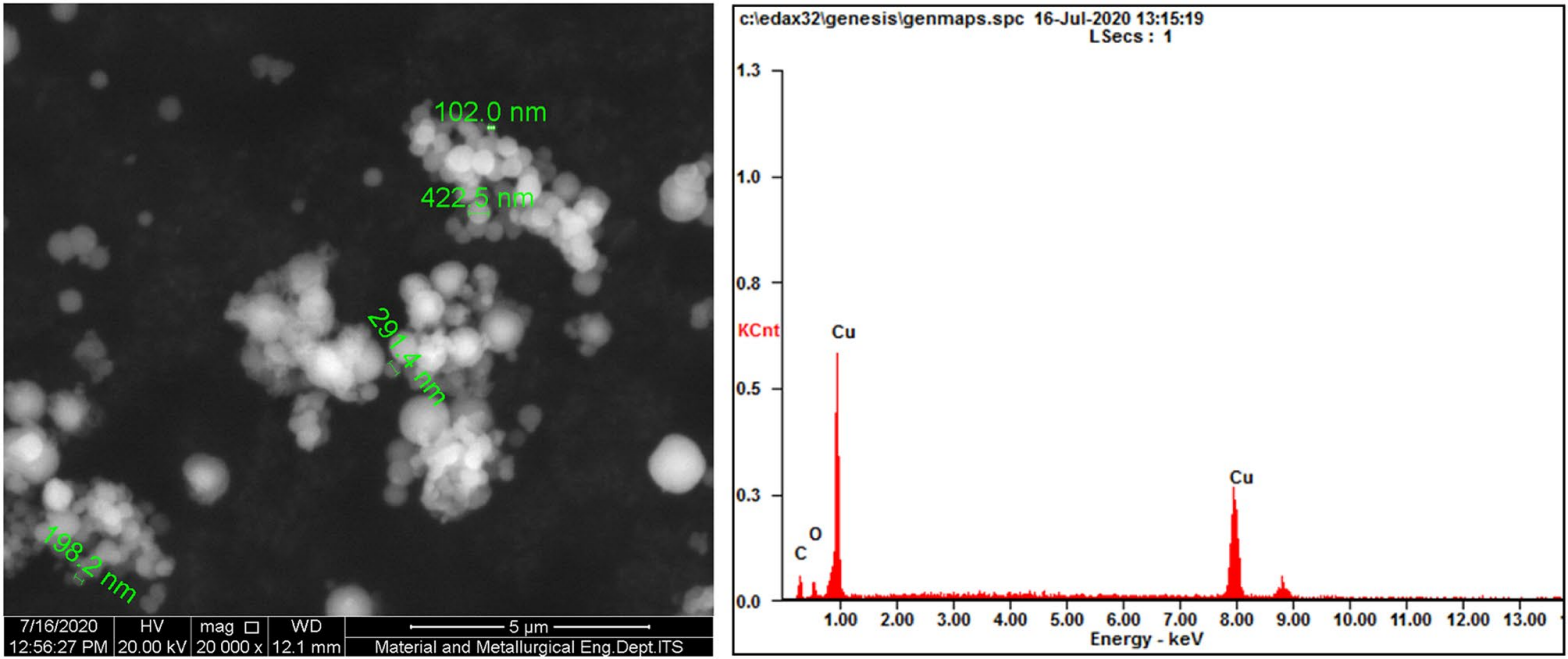
A SEM image of the CuNPs and an EDX spectrum of the spot particles. Left picture: the average size of the particles measured was 254 nm, ranging from 102 to 423 nm in the SEM image. Right graph: the percentage of Cu_2_O element composition on spot particles counted using EDX.

**Figure 2:**
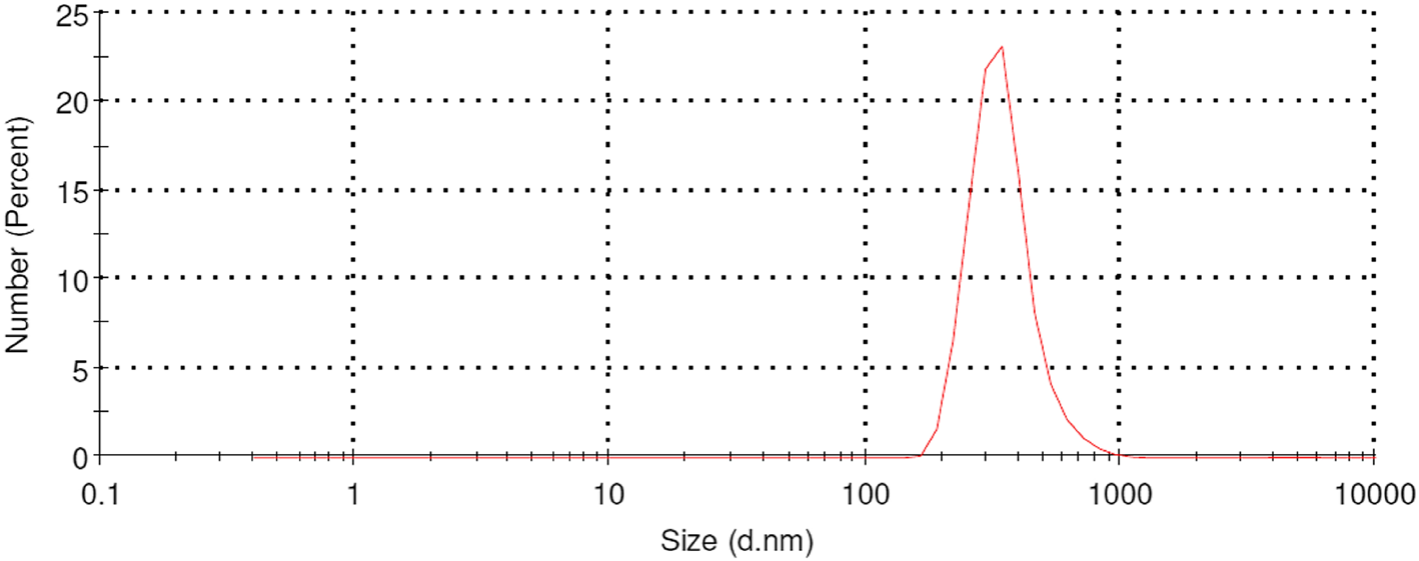
Particle size distribution of CuNP particles as measured by PSA. The mean size is 576 nm.

**Figure 3:**
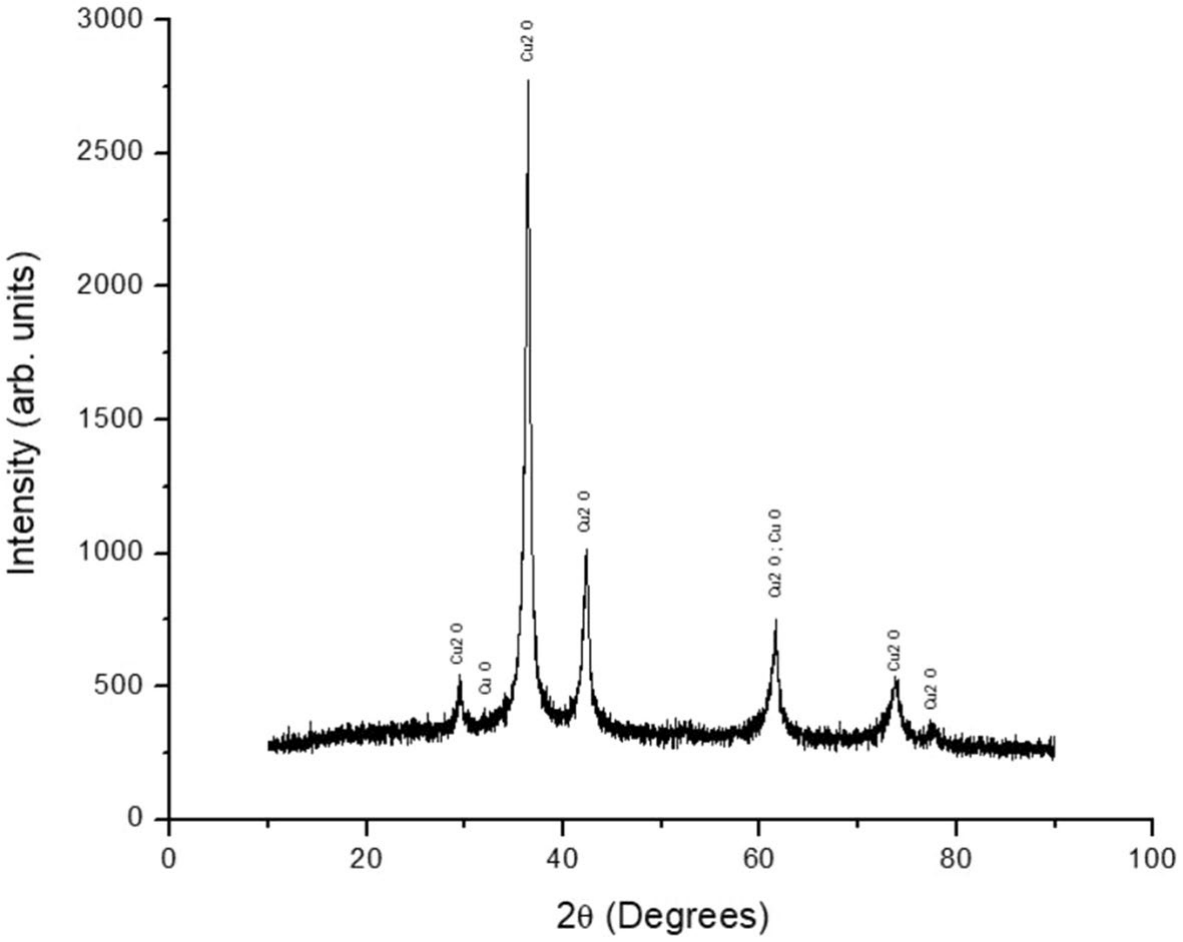
XRD analysis of the CuNPs/paint spray materials. There are two identified phases of copper oxide, cuprous oxide (Cu_2_O) and cupric oxide (CuO). The phase with the highest intensity and peak was Cu_2_O, which means that it is the most dominant phase on the synthesized coating. The peaks appear at multiple 2 theta degrees: 29.53°, 36.40°, 42.36°, 61.57°, 73.80°, and 77.67°. Some CuO phases were also detected at 2 theta 32.01° and 61.65°.

SEM was used to examine the morphology and size of the CuNPs as a result of Cu synthesis from CuSO_4_ to cuprous oxide (Cu_2_O). Figure 1 left picture shows an image obtained with 20,000 power of magnification. Typical spherical shapes of CuNPs tend to agglomerate. Most of the particles agglomerated to form larger sizes. The average size of the particles measured was 254 nm: the lowest and largest sizes were 102 nm and 423 nm, respectively. Figure 1 right graph shows the percentage of Cu_2_O element composition on spot particles counted using EDX. The size of the particles was further analyzed using PSA. As shown in Fig. 2, the average size of the mean was 576 nm by PSA. Unlike SEM, PSA tends to scan all particle sizes, including the agglomerated structures on the surface, thus expecting to obtain higher average particle sizes. During the analysis by PSA, the Cu_2_O was diluted with 50 mL of distilled water and analyzed at 25 °C with a 118 kcps count rate for 70 s. Figure 3 demonstrates the XRD results of the copper spray materials. There were two identified phases of Cu_2_O and cupric oxide (CuO). The phase with the highest intensity and peak was Cu_2_O, which means that it was the most dominant phase on the synthesized coating. The peaks appeared at multiple 2 theta degrees: 29.53°, 36.40°, 42.36°, 61.57°, 73.80°, and 77.67°. Some CuO phases were also detected at 2 theta 32.01° and 61.65°. The composition of the copper nanoparticles on our metal-coated surface is predominantly Cu_2_O, as shown in Fig. 3.

### Recovery of SARS-CoV-2 infectivity from CuNP/paint-coated surfaces

To obtain a rough idea of the time course for SARS-CoV-2 inactivation on the CuNP/paint-coated stainless steel surface, residual infectivity was measured at 0 h (collected immediately after exposure into a test tube), 4 h, and 24 h exposure in this preliminary experiment. The virus dose used was 10^4.1^ TCID_50_ in a volume of 20 µL. After 4 h, the infectivity decreased to an undetectable level, less than 10^0.8^, on the CuNP/paint surface, while it did not decrease substantially on the control surfaces of paint alone-coated and uncoated stainless steels. The infectivity diminished at 24 h in all groups.

Based on these results, different time schedules were employed in the next experiment for the CuNP/paint-coated and control surfaces to determine a more detailed time course of inactivation. To increase the dynamic range of response, a virus dose of 10 times more than the previous experiment was used. Residual infectivity and RdRP RNA copy number were measured at 0 min, 10 min, 30 min, 1 h, 2 h, and 4 h for the CuNP/paint-coated surface using a virus dose of 10^5.1^ TCID_50_, while they were measured at 0 min, 4 h, 8 h, 12 h, 18 h, and 24 h for the paint alone-coated and uncoated surfaces. As a control, the same virus suspension in a plastic test tube was placed in the same place, sampled on the same time schedule, and assayed for those two parameters.

**Figure 4:**
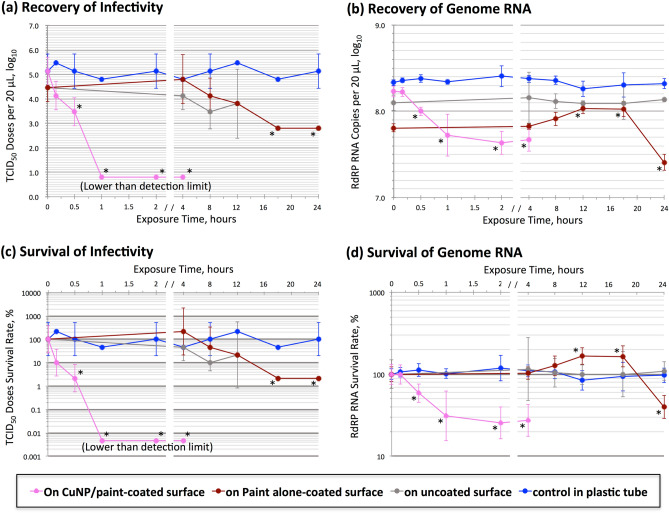
Inactivation of SARS-CoV-2 infectivity on CuNP/paint-coated stainless steel surfaces. An aliquot of 20 µL of virus dilution was applied on the test surface and covered by a circular glass slip of 1.13 cm in diameter foaming a cylindrical shape of 1 cm^2^ base area by 0.2 mm height and placed inside a class II safety cabinet at 24 °C with relative humidity of 40–50% for various times. Then, the virus suspensions were retrieved in test tubes, titrated for infectivity in cell culture, and subjected to RT-PCR for RdRP RNA to estimate the copy number of the genomic RNA. (**a**) Recovery of infectivity. Infectivity doses of TCID_50_ per 20 µL in log10 were plotted for exposure times. (**b**) Recovery of genome RNA. RdRP RNA copy numbers per 20 µL in log10 were plotted. (**c**) Survival of infectivity. The percentages of the recovered infectivity at various times to that at 0 h were plotted. (**d**) Survival of genome RNA. The percentages of the recovered RdRP RNA copy number at various times to that at 0 h were plotted. (**a**–**d**) Pink circle and connecting line: on CuNP/paint-coated stainless steel surface; Russet: on paint-coated surface; Gray: on uncoated surface; Brue: control in plastic tube. *: The decrease or increase from the value at 0 min was statistically significant (*P* < 0.05).

Figure 4a shows the time course of changes in residual infectivity. On the CuNP/paint-coated surface, the infectivity decreased from 10^5.1^ at 0 min to 10^4.1^ (1.0 log10-fold reduction, statistically insignificant: *P* = 0.101) at 10 min, to 10^3.5^ (1.6 log10-fold reduction, significant: *P* = 0.024) at 30 min, and to an undetectable level of less than 10^0.8^ (more than 4.3 log10-fold reduction, significant: *P* = 0.006; negative samples are denoted with a TCID_50_ of 10^0.8^, which was the limit of detection) at 1 h, 2 h, and 4 h. On the paint alone-coated and uncoated surfaces, the infectivity of 10^4.5^ at 0 min decreased insignificantly at 4 h, 8 h, and 12 h. It decreased to 10^2.8^ (1.7 log10-fold reduction, significant: *P* = 0.038) at 18 h and 24 h. In the control plastic tube, the virus titer remained the same level between 10^4.8^ and 10^5.5^ at 0 min to 24 h.

### Recovery of SARS-CoV-2 genomic RNA from CuNP/paint-coated surfaces

Figure 4b shows the time course of changes in recovered RdRP RNA copy number. On the CuNP/paint-coated surface, the copy number decreased from 10^8.268^ at 0 min to 10^8.223^ (0.045 log10-fold reduction, insignificant: *P* = 0.920) at 10 min, to 10^8.00^ (0.23 log10-fold reduction, significant: *P* = 0.007) at 30 min, to 10^7.72^ (0.51 log10-fold reduction, significant: *P* = 0.025) at 1 h, to 10^7.63^ (0.60 log10-fold reduction, significant: *P* = 0.002) at 2 h, and to 10^7.67^ (0.56 log10-fold reduction, significant: *P* = 0.003) at 4 h. For the paint alone-coated surface, the copy number of 10^7.81^ at 0 min slightly increased at 4 h and 8 h, significantly increased to 10^8.03^ at 12 h (*P* = 0.006) and 18 h (*P* = 0.017), then significantly decreased to 10^7.41^ at 24 h (*P* = 0.003). The copy numbers of 10^8.10^ on the uncoated surface and 10^8.33^ in the control plastic tube at 0 min were not significantly changed from 0 min to 24 h.

### Recovery rates of infectivity and genomic RNA

Recovery rates of infectivity and RdRP RNA for each surface were calculated by dividing the recovered infectious dose or RdRP RNA copy number at 0 min by that in the control plastic tube. Whereas recoveries of RdRP RNA were 79% (insignificant: *P* = 0.066), 30% (significant: *P* = 0.0001), and 58% (significant: *P* = 0.012), those of infectivity were 100%, 22% (insignificant: *P* = 0.230), and 22% (insignificant: *P* = 0.230) for the CuNP/paint-coated, paint alone-coated, and uncoated stainless steel surfaces, respectively. It was considered that the unrecovered infectivity and RNA were attached to each surface, most likely by electrostatic attractive force. The unrecovered rates were lowest with the CuNP/paint-coated surface (0% infectivity and 21% RdRP RNA) and highest with the paint alone-coated surface (78% infectivity and 70% RdRP RNA).

### Survival rates of infectivity and genomic RNA of SARS-CoV-2 on CuNP/paint-coated surfaces

To analyze the relationship between the reduction in infectivity and that of genome RNA copy number on the tested surfaces, the survival kinetics were compared, as shown in Fig. 4c, d. The survival rate was calculated by dividing the recovered infectious dose or RdRP RNA copy number by that at 0 min. On the CuNP/paint-coated surface, the infectivity survival rate was 10% (the reduction was statistically insignificant; *P* = 0.101) at 10 min, 2.2% (significant; *P* = 0.024) at 30 min, and less than 0.005% (significant; *P* = 0.006) at 1 h, 2 h, and 4 h. In other words, the infectivity inactivation rate on the CuNP/paint-coated surface was 90% at 10 min, 97.8% at 30 min, and more than 99.995% at 1 h, 2 h, and 4 h; the half-life was calculated to be 0.16 h. On the paint alone-coated and uncoated surfaces, the infectivity survival rates were not significantly changed (*P* > 0.05) at 4 h, 8 h, and 12 h and significantly reduced to 2.2% (*P* = 0.038) at 18 h and 24 h. There was no significant reduction in the survival rate in the control tube even at 24 h.

The survival rate of RdRP RNA copy number on the CuNP/paint-coated surface was 99% (the reduction was statistically insignificant; *P* = 0.920) at 10 min, 59% (significant; *P* = 0.007) at 30 min, 31% (significant; *P* = 0.025) at 1 h, 25% (significant; *P* = 0.002) at 2 h, and 27% (significant; *P* = 0.003) at 4 h. It was indicated that approximately 30% of RdRP RNA was resistant to degradation on the CuNP/paint-coated surface. On the paint alone-coated surface, the survival rate increased to 104% (insignificant; *P* = 0.632) at 4 h, to 127% (insignificant; *P* = 0.112) at 8 h, to 168% (significant; *P* = 0.006) at 12 h, to 165% (significant; *P* = 0.017), and afterward decreased to 40% (significant; *P* = 0.003) at 24 h. The survival rate was unchanged on the uncoated surface and in the control plastic tube. The survival kinetics of RdRP copy number was different from that of infectivity, especially on the CuNP/paint-coated and paint-alone-coated surfaces.

## Discussion

We successfully established a method to synthesize CuNPs of Cu_2_O with a mean size of 254 nm from copper sulfate. Through the two-step reduction method, the uniformity of the copper nanoparticles was successfully synthesized. Wen et al.^18^ proved this prominent feature of the method using glucose and sodium hypophosphite as the reductors, whereas we used ascorbic acid as the second reductor. The study of Behzadinasab et al. used a similar approach to our study, which incorporated Cu_2_O oxide nanoparticles in the form of film coatings^20^. In our study, we advocated the usage of a spray method to apply CuNPs in consideration of its practicality, portability, and wide availability.

Our results showed that SARS-CoV-2 infectivity was inactivated 90% by 10 min, 97.8% by 30 min, and more than 99.995% by 60 min exposure to the CuNP/paint-coated surface (Fig. 4c), and the half-life was 0.16 h. In contrast, the virus infectivity was inactivated insignificantly in 60 min and by 97.8% in 18 h exposure to paint alone-coated and uncoated stainless steel surfaces. The inactivation rate on the CuNP/paint-coated surface was approximately 36-fold faster than that of the control surfaces. Van Doremalen et al*.* reported that the half-life of SARS-CoV-2 on the surface of copper metal remnants was 0.774 h^4^. An approximately 5 times shorter half-life for the CuNP/paint-coated surface, that is, 0.16 h, indicates that CuNPs are 5 times more efficient than copper metal to inactivate SARS-CoV-2 infectivity. Hutasoit et al*.* also reported that copper powders with particle sizes ranging from 5 to 60 µm coated on in-use steel parts could inactivate SARS-CoV-2 infectivity by 96% in 2 h^21^, whereas CuNPs/paint inactivated SARS-CoV-2 infection by 97.8% after 30 min of exposure. The reduction rate after 1 h on the surface of Cu_2_O particles of a mean size of 5.5 µm bound with polyurethane was approximately 99.9%^20^. In our study, the mean size of the CuNPs was 254 nm in diameter, 20-fold smaller than that, and the available surface area dramatically increased; thus, CuNPs had a one-order higher inactivation ability of more than 99.995% after 1 h of exposure.

The survival rate of RdRP RNA copy number on the CuNP/paint-coated surface decreased to 31% during 1 h of exposure. However, no further decrease occurred after 2 h and 4 h of exposure, suggesting that approximately 30% of RdRP RNA was resistant to degradation on the CuNP/paint-coated surface (Fig. 4d). No such resistant fraction was observed for infectivity, which indicated that the survival kinetics of RdRP RNA copy number was not correlated with that of infectivity. The virus was propagated using Vero monolayer cells and harvested after 2 d incubation when most of the cells died. Thus, the obtained virus pool possibly contained viral RNAs that were released from the dead cells without being incorporated in the virus particles. Therefore, the degradation-sensitive fraction of 70% was not associated with infectivity. On the other hand, the degradation-resistant fraction of 30% was most likely inside the virus particle and protected against attack of antiviral substances that might be produced on the CuNP/paint-coated surface. Instead, the inactivation of 90% at 10 min, 97.8% at 30 min, and 99.995% at 1 h of infectivity can be explained by inactivation of the function(s) of virus spikes that reside on the surface of the virus particle and play essential roles in infection, virus attachment to the host cell and virus envelope-cell membrane fusion. Fujimori et al*.* reported that Cu(I) iodide nanoparticles inactivated the influenza A H1N1 pandemic 2009 strain, in which hydroxyl radicals of reactive oxygen species were produced and degraded hemagglutinin and neuraminidase viral surface proteins^22^. The viral envelope of the lipid bilayer can also be a target of reactive oxygen species^23^. A similar mechanism(s) may be involved in the inactivation of SARS-CoV-2 on the CuNP/paint-coated surface.

The RdRP RNA survival kinetics on the paint-alone-coated surface showed an irregular pattern; the recovered copy number significantly increased at 12 h and 18 h to up to 168% of the initial number at 0 h and then decreased at 24 h to 40% (Fig. 4d), while the infectivity decreased to 22% at 12 h and to 2.2% at 18 h and 24 h (Fig. 4c). This could be explained by the high binding rate of infectious virus and RdRP RNA with the paint-alone-coated surface (78% infectivity and 70% RdRP RNA). The majority of the bound RNAs may be degradation-sensitive RNAs. Our RT-PCR test for RdRP RNA measured copy number containing a 100-nt sequence of RdRP gene in the whole genome of 29,903-nt length. When attached RNA of up to the full length becomes degraded to shorter fragments on the surface, some fragments are released from the surface to the recovery fluid. The increase at 12 h and 18 h could occur by this mechanism. At 24 h, the degradation process proceeded inside the 100-nt sequence and consequently decreased the recovery rate.

From our results, the application of CuNPs/paint coating on public or hospital facilities and other commonly touched areas is expected to be beneficial. However, further study is needed to provide more details regarding the inactivation mechanisms, their effective duration, and their cytotoxicity and environmental derangement.

## Conclusions

We successfully established a method to synthesize CuNPs of Cu_2_O with a size of 254 nm from copper sulfate. The CuNP/paint-coated surface inactivated more than 99.995% of the infectivity of SARS-CoV-2 after 1 h of exposure, although further study is needed to elucidate the inactivation mechanisms. The application of CuNPs/paint coating to public or hospital facilities and other commonly touched areas is expected to be beneficial.

## Data Availability

The datasets used and/or analyzed during the current study are available from the corresponding author upon reasonable request.
