# Author Correction: Synthesis and assessment of copper-based nanoparticles as a surface coating agent for antiviral properties against SARS-CoV-2

**DOI:** 10.1038/s41598-022-09898-z

**Published:** 2022-04-05

**Authors:** Agung Purniawan, Maria Inge Lusida, Royan Wafi Pujiyanto, Aldise Mareta Nastri, Adita Ayu Permanasari, Alfonsus Adrian Hadikusumo Harsono, Nur Hafidzah Oktavia, Sigit Tri Wicaksono, Jezzy Renova Dewantari, Rima Ratnanggana Prasetya, Krisnoadi Rahardjo, Mitsuhiro Nishimura, Yasuko Mori, Kazufumi Shimizu

**Affiliations:** 1 Department of Materials and Metallurgical Engineering, Institut Teknologi Sepuluh Nopember, Surabaya, Indonesia; 2 Research Center for Advanced Materials and Nanotechnology, Institut Teknologi Sepuluh Nopember, Surabaya, Indonesia; 3 Institute of Tropical Disease, Indonesia-Japan Collaborative Research Center for Emerging and Re-Emerging Infectious Diseases, Universitas Airlangga, Surabaya, Indonesia; 4 Center for Infectious Diseases, Kobe University Graduate School of Medicine, Kobe, Japan

Correction to: *Scientific Reports,* 10.1038/s41598-022-08766-0, published on 22 March 2022

The original version of this Article contained errors in the Materials and methods section, under the subheading ‘RT-PCR’,

“The reaction conditions were as follows: step 1, reverse transcription for 15 min at 50 °C; step 2, initial denaturation for 3 min at 95 °C; step 3, 5 cycles of preamplification for 5 s at 95 °C and 40 s at 60 °C to activate Taq polymerase; and step 4, 40 cycles of amplification for 5 s at 95 °C and 40 s at 60 °C.”

now reads:

“The reaction conditions were as follows: step 1, reverse transcription for 15 min at 50 °C; step 2, initial denaturation for 3 min at 95 °C to inactivate reverse transcriptase and to activate Taq polymerase; and step 3, 45 cycles of amplification for 5 s at 95 °C and 40 s at 60 °C.”

“RT-PCR was performed using a LightCycler 480 and its software (Roche, Switzerland).”

now reads:

“RT-PCR was performed using an Applied Biosystems 7500 Fast and its software (Applied Biosystems, Massachusetts, USA).”

Additionally, this Article contained a repeated error in the word “steel” that was incorrectly given as “steal”. This error has been corrected throughout the Article.

Finally, the original version of this Article contained an error in the order of the Figures, where Figures 2 and 3 were published as Figures 3 and 2.

The original Figures 2 and 3 and accompanying legends appear below.

The original Article has been corrected.

**Figure 2:**
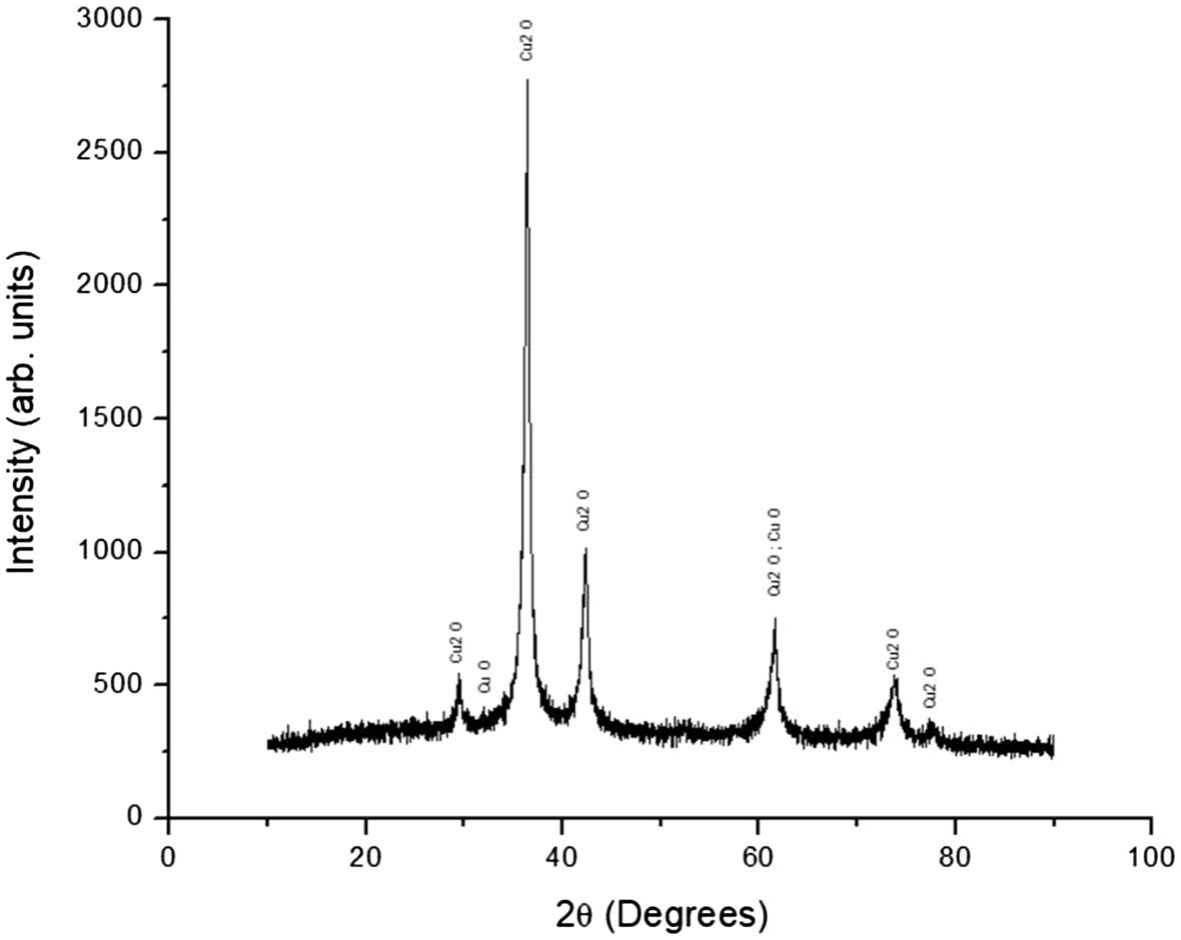
XRD analysis of the CuNPs/paint spray materials. There are two identified phases of copper oxide, cuprous oxide (Cu_2_O) and cupric oxide (CuO). The phase with the highest intensity and peak was Cu_2_O, which means that it is the most dominant phase on the synthesized coating. The peaks appear at multiple 2 theta degrees: 29.53°, 36.40°, 42.36°, 61.57°, 73.80°, and 77.67°. Some CuO phases were also detected at 2 theta 32.01° and 61.65°.

**Figure 3:**
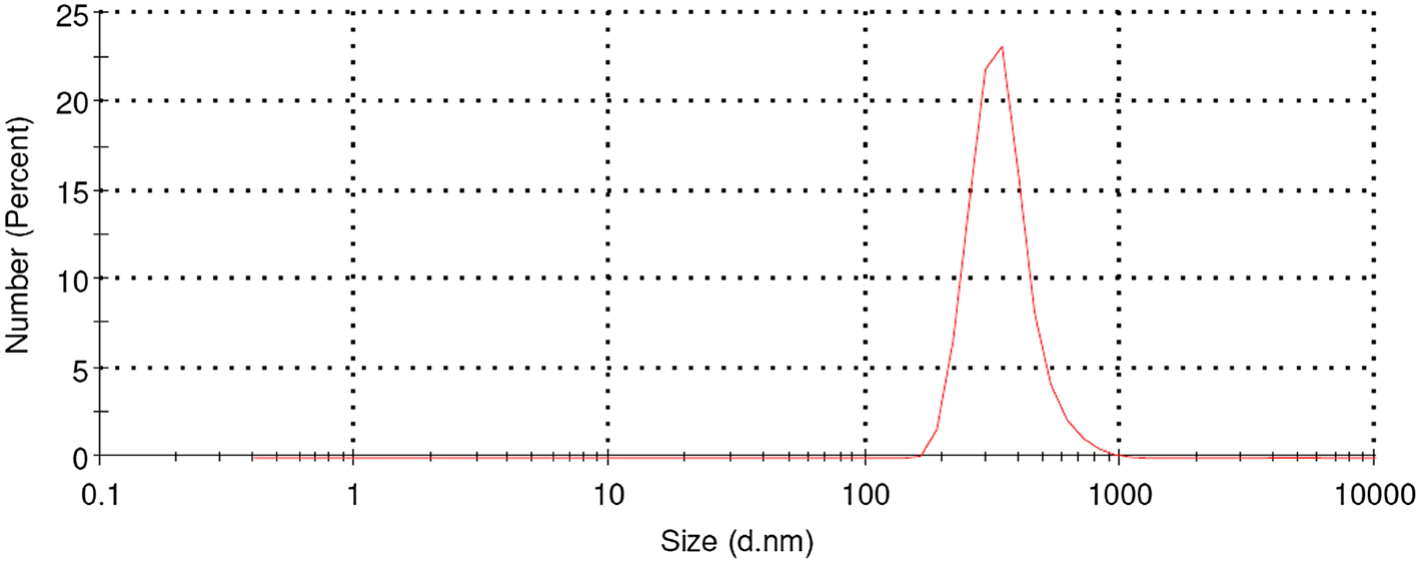
Particle size distribution of CuNP particles as measured by PSA. The mean size is 576 nm.

